# A Longitudinal Transdisciplinary Approach for Autism Spectrum Disorder

**DOI:** 10.3390/children12091272

**Published:** 2025-09-22

**Authors:** Aline Kabarite, Glória Maria Marques Ferreira, José Carlos Pitangueira, Rayana de Souza Arimatéa, Renata da Costa Rebello de Mendonça, Roberta Sousa Marcello, Thais Giudice Schulz, Rudimar dos Santos Riesgo, Kamila Castro

**Affiliations:** 1 Instituto Priorit, Departamento de Fonoaudiologia, Rio de Janeiro 22631-003, Brazil; alinekabarite@institutopriorit.com.br (A.K.); renatamendonca@institutopriorit.com.br (R.d.C.R.d.M.); 2 Instituto Priorit, Departamento de Psicologia, Rio de Janeiro 22631-003, Brazil; gloriamarques@institutopriorit.com.br (G.M.M.F.); rayanaarimatea@institutopriorit.com.br (R.d.S.A.); robertamarcello@institutopriorit.com.br (R.S.M.); 3 Instituto Priorit, Departamento Pesquisa, Desenvolvimento e Inovação, Rio de Janeiro 22631-003, Brazil; jcpitangueira@institutopriorit.com.br; 4 Instituto Priorit, Departamento de Terapia Ocupacional, Rio de Janeiro 22631-003, Brazil; thaisgiudices@institutopriorit.com.br; 5 Unidade de Neurologia Infantil do Hospital de Clínicas de Porto Alegre, Porto Alegre 90035-903, Brazil; rriesgo@hcpa.edu.br; 6 Grupo de Pesquisa Translacional em Transtorno do Espectro Autista, Universidade Federal do Rio Grande do Sul, Porto Alegre 90610-264, Brazil

**Keywords:** autism spectrum disorder, transdisciplinary care, family participation, treatment outcome

## Abstract

**Highlights:**

- The implementation of a flexible, team-based, and personalized care model promoted improvements in functioning and reduced associated characteristics in children with ASD over a 12-month period.
- Greater family involvement led to better outcomes, highlighting the importance of caregiver involvement in intervention.

**What are the main findings?**

- A transdisciplinary, family-centered care model led to significant improvements in functioning, behavior, and support needs in children and adolescents with ASD.
- Most participants reached high functional levels after 12 months, with greater progress linked to stronger family involvement.

**What is the implication of the main finding?**

- Personalized and collaborative interventions are effective in promoting developmental gains in ASD care.
- Family participation should be prioritized as a key component of successful long-term intervention outcomes.

**Abstract:**

**Background/Objectives**: Autism Spectrum Disorder (ASD) presents complex developmental challenges that require coordinated, individualized interventions. This study aimed to evaluate the effectiveness of a transdisciplinary, family-centered approach in improving clinical and functional outcomes in children and adolescents with ASD. **Methods**: A longitudinal study was conducted with 53 participants aged 2 to 16 years, all with confirmed ASD diagnoses. Assessments were performed at baseline, 6 months, and 12 months. Participants received personalized, evidence-based interventions provided by a multidisciplinary team working within a transdisciplinary model. Therapies were delivered individually and in groups, with flexible intervention phases tailored to each participant’s evolving needs. Outcomes were measured using the Clinical Global Impression (CGI), Global Assessment of Functioning (GAF), and the Aberrant Behavior Checklist (ABC). **Results**: Clinical and functional improvements were observed over the 12-month period. Most participants reached high functional levels by the end of the study. Caregivers reported notable reductions in support needs, while therapist ratings confirmed more moderate improvements. Decreases in behavioral challenges, sensory difficulties, and sleep disturbances were observed, alongside gains in adaptability and functional play. Greater family involvement was associated with more favorable outcomes. **Conclusions**: A transdisciplinary, family-centered intervention model was beneficial in supporting developmental progress in children and adolescents with ASD. The findings highlight the importance of flexible, team-based care and emphasize the critical role of family engagement in achieving positive long-term outcomes.

## 1. Introduction

Autism spectrum disorder (ASD) comprises a diverse set of neurodevelopmental conditions, marked by early challenges in social communication and repetitive behaviors and interests [1]. Worldwide, ASD prevalence is estimated at approximately 1%, and recent reports indicate rising diagnosis rates [2]. While ASD diagnostic criteria include two primary domains—social communication and restricted behaviors—the presentation of characteristics is highly variable, necessitating nuanced, individualized diagnostic assessments. This variability also reflects the presence of individual strengths and interests that are frequently underrepresented in clinical descriptions of ASD [3]. Recognizing these characteristics is essential in both clinical practice and research, as it contributes to more nuanced and inclusive characterizations of individuals with ASD. Despite advances in understanding the genetic underpinnings of ASD, no single biomarker has been identified, underscoring the complexity of this neurobiologically rooted condition [4].

ASD support needs vary, which the DSM-5-TR addresses by categorizing ASD into three levels based on required support. These levels guide personalized intervention plans, recognizing that support requirements differ across daily living, social, and behavioral domains [1]. Beyond support levels, behavioral and psychiatric conditions in individuals with ASD are an important area of study, with prevalence estimates ranging widely depending on diagnostic criteria, access to services, and sociocultural context. For example, in some cultural contexts, behavioral difficulties such as hyperactivity may be construed as a normative or, conversely, stigmatized, thereby hindering timely formal diagnosis. Such sociocultural determinants contribute to cross-population variability in the identification and reporting of comorbidities. Frequently reported co-occurrences include anxiety, mood disorders, attention-deficit/hyperactivity disorder (ADHD), obsessive–compulsive disorder (OCD), and oppositional defiant presentations [4,5].

Early diagnosis and intervention are essential, as research suggests that comprehensive, behavior-based interventions can enhance social communication and reduce anxiety and aggression in children with ASD. A multidisciplinary approach is critical, given the wide-ranging needs of individuals with ASD. Collaboration across fields—including psychology, speech therapy, occupational therapy, and medical professionals—allows for integrated care and has been shown to improve outcomes by tailoring interventions to individual needs [6]. It is important to emphasize that a multidisciplinary intervention does not necessarily imply a high-intensity schedule. Interventions should always be adjusted to respect the individual’s well-being, adopting ethical, person-centered approaches that prioritize engagement and autonomy. Recent discussions have raised concerns about the potential emotional impact of overly rigid or prolonged interventions, such as fatigue and distress [7]. These ethical considerations and the risk of therapy-related fatigue should be taken into account when designing interventions, reinforcing the importance of flexibility, individualization, and respect for the child’s emotional well-being.

Multidisciplinary intervention offers a strong foundation for autism, which follows the demands of this condition, which is often complex and for a lifetime. In a multidisciplinary model, professionals from different disciplines work in parallel, each addressing the individual’s needs from their own specialized perspective. Research has shown that such collaboration can significantly improve outcomes for individuals with ASD by providing a more tailored intervention [8]. Additionally, a multidisciplinary team can offer a wider range of resources and support for families, making the intervention process more efficient and effective. An integrated approach not only addresses the diverse experience associated with autism but also enhances their overall quality of life [9].

Due to the growing complexity of autism and the number of different professions that work with these individuals, there is a need for improved interprofessional collaboration using a multidisciplinary approach. According to the World Health Organization [10] this can be defined as having different professions, coming together to work towards a common goal for patients, families, caregivers, and communities to deliver the highest quality of care. A few studies are reported in the literature describing the results from an intervention applied by a multidisciplinary team for ASD. This differs from transdisciplinary approaches, in which professionals collaborate across disciplines to develop shared frameworks and integrated strategies [11]. Collaboration—especially when it moves toward transdisciplinary practice—can be a key factor in efforts that aim to increase how effective services are for children on the autism spectrum [9].

Transdisciplinarity in health care involves transcending traditional disciplinary boundaries to address complex health issues. This approach integrates knowledge from various fields, including natural, social, and health sciences, and emphasizes collaboration among diverse stakeholders such as patients, families, and communities [8,12,13]. In the context of autism, a transdisciplinary perspective may also benefit from including autistic individuals in the development of strategies. When scientific expertise is integrated with lived experience, care models can become more attuned to individual needs and perspectives, supporting approaches that are both person-centered and respectful of neurodiversity.

Additionally, various factors have been identified that improve a child’s success in intervention. Family involvement is one of these, studies indicate that family-centered approach to these interventions has been shown to lead to improved outcomes. In many cases, parental involvement in intervention covers a broad range of activities, from parent training and homework routines, even participation in intervention design and implementation [14]. However, while this engagement can enhance intervention effectiveness, it may also place emotional and logistical strain on caregivers, especially when expectations exceed available support or resources. Acknowledging and addressing the emotional burden on families is essential for ensuring sustainable, ethical, and supportive care models. Studies have reported increased levels of stress, anxiety, and even symptoms of depression among caregivers of children with ASD, especially when support networks are limited [15,16].

Considering the multifaceted aspects related to ASD, this study aims to evaluate the effects of a transdisciplinary, family-centered intervention model on global functioning, clinical support needs, and maladaptive behaviors in children and adolescents with ASD over a 12-month period. The study draws on a clinical sample from a specialized service context, offering insights into the practical implementation and relevance of the intervention in real-world settings.

## 2. Materials and Methods

### 2.1. Study Design

This is a longitudinal study with a one-year follow-up, designed to assess clinical and functional progress in children and adolescents diagnosed with ASD. To ensure consistent baseline measures and account for possible cohort effects, recruitment was conducted in 10 waves over a 16-month period. To address the potential for data fragmentation resulting from individualized and variable intervention pathways, standardized outcome measures were administered at fixed time points (baseline, 6 months, and 12 months) for all participants. This consistent assessment schedule allowed for comparability of clinical and functional outcomes across the sample, even in the dynamic treatment trajectories.

### 2.2. Sociodemographic Data Collection

Sociodemographic variables, including age, sex, caregiver education level, and family income, were collected at baseline and updated as needed during follow-up. Socioeconomic status was classified using the Brazilian Economic Classification Criterion (Critério de Classificação Econômica Brasil—CCEB) developed by the Brazilian Association of Research Companies (ABEP), a widely accepted tool for stratifying populations based on income, education, and material resources. This classification was primarily used to characterize the sample.

### 2.3. Participants

Participants were children and adolescents aged 2 to 16 years with a confirmed diagnosis of ASD. All diagnoses were made by external licensed child psychiatrists or pediatric neurologists, based on DSM criteria. Diagnostic documentation was verified through medical records prior to inclusion. All participants were enrolled in at least one individual therapy program at a private clinic specializing in neurodevelopmental profiles. Secondary diagnoses (e.g., ADHD, anxiety, intellectual disability) were not used as exclusion criteria and were documented for descriptive purposes.

### 2.4. Training of Professionals

All professionals involved in the study received standardized training to ensure consistent data collection and intervention delivery. The training included an immersion phase in which each professional observed and shadowed experienced staff across disciplines for one week. Additionally, to monitor intervention fidelity, meetings were conducted to review individual treatment plans, track progress, and ensure alignment with the structured methodology. Supervisors also conducted regular structured observations informed by the intervention model. These evaluations focused on key elements such as the clarity of therapeutic objectives in each phase, integration across disciplines, active family participation, quality of communication among professionals, and respect for each child’s individual rhythm and developmental profile. Written and verbal feedback was provided during supervision meetings, and the results were used to support team reflection and guide necessary adjustments to therapeutic strategies.

Assessments were conducted by a team of licensed professionals, including psychologists, occupational therapists, and speech therapists, all with clinical experience in ASD. All assessors had prior experience working with children and adolescents with ASD.

In this study, the lead therapist is the professional responsible for coordinating the transdisciplinary care provided to the patient.

### 2.5. Setting and Interventions

The research site has professionals specialized in autism intervention in the areas of speech therapy, psychology, psychopedagogy, occupational therapy, music therapy, and family therapy. Additionally, group activities can be carried out with patients such as capoeira, theater, judo, yoga, dance, psychomotricity, and social skills training. All interventions were conducted in a clinical setting. No school-based, home-based, or telehealth services were included in this study.

The Priorit Methodology encompasses multidisciplinary teams with transdisciplinary action, that is, a team that works jointly in constant communication to create new strategies based on scientific evidence that integrates and moves beyond demand-specific approaches to address a common aim. The intervention model used in this study was not based on standardized models. Therapeutic planning was adapted to each child’s developmental profile and evolving needs. The model incorporates principles from developmental and humanized care approaches, emphasizing emotional engagement, family involvement, and real-world adaptability.

The combined interventions available at the time of the research were not the same for all patients; each one carried out the interventions according to their demands. The distribution of the prevalence of patients in each activity is included in Appendix A, Table A1.

**Figure 1:**
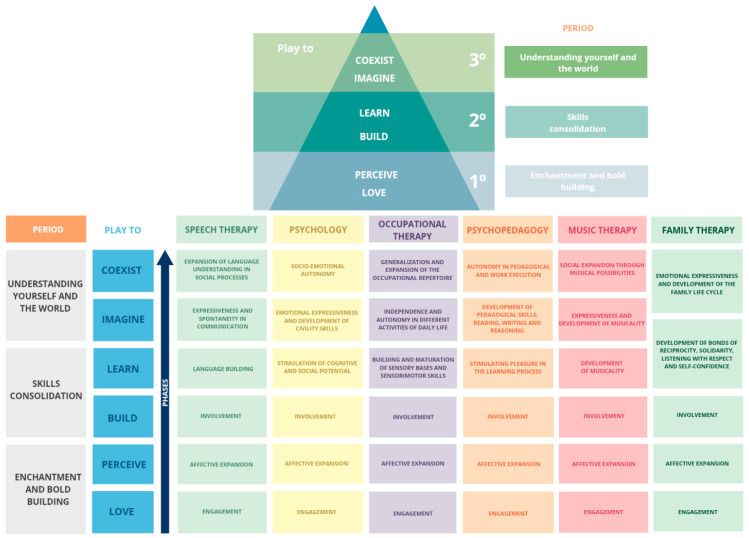
Priorit Methodology. This figure presents the structure of the Priorit Methodology, which organizes therapeutic processes into three broad developmental periods (Enchantment and Bold Building, Skills Consolidation, and Understanding Yourself and the World), subdivided into six phases (Love, Perceive, Build, Learn, Imagine, and Coexist). Each phase includes objectives distributed across multiple disciplines—speech therapy, psychology, occupational therapy, psychopedagogy, music therapy, and family therapy—reflecting a transdisciplinary and family-centered approach. While the model offers a comprehensive and flexible structure for intervention planning, it was specifically developed for implementation within a transdisciplinary team, and some variation in internal consistency across phases is expected.

Therapeutic interventions followed the same protocol until achieving the specific level (Figure 1). All patients went through all phases of intervention. It is important to highlight that these periods and phases do not have a specific time, they are dynamic and can often overlap. The patient’s advancement to the next phase always depends on the response to intervention and the objectives achieved and generalized. One of the main characteristics of this methodology is that, through the application of evidence-based practices, it aims not only at therapeutic evolution but also at creating bonds and working in the affective sphere.

### 2.6. Procedures and Outcomes Measures

Sociodemographic variables were collected at the time of baseline and subsequently updated as necessary. Clinical data were collected at each follow-up point.

For clinical evaluation, the following questionnaires were applied:

Questionnaire: A questionnaire with questions stratified by age and area (behavioral aspects, anxiety, socialization, basic and instrumental activities of daily living, communication and language, motor development, sensory perception, academic skills, and family aspects). This is a questionnaire developed by a multidisciplinary team, aimed at assessing the patient based on their current capacities and demands across different scenarios beyond just the therapeutic setting. It is administered to the patients’ caregivers.

Clinical Global Impression (CGI): The clinical global impression rating scales are measures of symptom severity, treatment response, and the efficacy of intervention in treatment studies of patients with mental conditions. It is a brief 3-item observer-rated scale that allows tracking symptom changes [17].

Global Assessment Functioning (GAF): This is a scoring system that mental health professionals use to assess how well an individual is functioning in their daily lives. This scale was once used to measure the impact of psychiatric illness on a person’s life and daily functional skills and abilities. The scores range from 0 to 100, with 100 representing superior functioning [18].

Aberrant Behavior Checklist (ABC): Consists of 58 questions across 5 different domains: irritability, social withdrawal, stereotypic behavior, hyperactivity/noncompliance, and inappropriate speech. The rater has to answer each of the 58 questions using a 4-point Likert scale [19,20].

### 2.7. Statistical Analysis

Statistical analysis of the data was performed using the SPSS 25.0 (SPSS Inc., Chicago, IL, USA) program. The normality of numerical variables was determined by the Kolmogorov–Smirnov test. Numerical variables were summarized as means (±standard deviations). Categorical variables were given as numbers and percentages. When comparing numerical variables between the two groups, the Mann–Whitney U test was used because the variables did not show a normal distribution. The chi-square test was used when comparing categorical variables. The correlation between the measurement variables was compared with the Pearson correlation test, and their correlation coefficients were calculated. The statistical significance level was taken as 0.05 for each test. Although no formal statistical control for confounding variables (e.g., SES, comorbidities) was conducted, standardized clinical instruments were applied consistently at three different moments across all participants. This repeated-measures structure allowed for within-subject comparisons over time and helped reduce potential measurement bias related to timing or evaluator variability.

### 2.8. Ethical Aspects

The protocol of the present study was approved by the Ethics and Research Committee of the Research and Postgraduate Group of the Hospital de Clínicas de Porto Alegre (Protocol number 95635618.6.0000.5327). Additionally, the researchers declare in this study that they complied with the terms of the Declaration of Helsinki, from its elaboration and its data disclosure. The informed consent process was not treated as a one-time event but as a continuous, dialogical practice. This ongoing consent approach reinforced transparency and trust, ensuring that participants remained voluntarily engaged and fully aware of their therapeutic journey.

## 3. Results

A total of 53 patients were included (67 initially enrolled), reflecting typical attrition over the course of longitudinal follow-up. The mean age was 9.0 ± 3.92 years old, and 80.6% of the samples was male. There were no baseline differences in sociodemographic, prenatal or birth data during follow-up (Table 1).

Regarding clinical aspects, most patients began their investigation into the identification of autism due to communication differences or speech delay (55.7%); the average age of onset of characteristics was 3.50 ± 2.02, while the average age of diagnosis was 4.03 ± 1.56.

Most patients performed more than one type of intervention (89%), and approximately 60% of patients performed at least one group activity. In the study sample, younger patients performed a greater number of therapies at all stages of the collection date.

There was a correlation between the highest economic level and the fact of carrying out more than two therapies (*p* = 0.032, *r* = 0.0678). However, this finding may be influenced by the characteristics of the private clinical setting and should be interpreted in light of that context. The majority of primary caregivers participating in the research were mothers (approximately 65%), and this data did not change over the course of a year. When evaluating the level of education of parents, most fathers had a graduate degree; however, the highest percentage at the postgraduate level was that of mothers (10%), while only 3% of fathers reached this educational level.

More than 60% of families started searching for diagnostic investigation because their child had more than one characteristic related to autism, such as, differences in communication or speech delay (53.7%) or behavioral difficulties (35.8%).

Most of the patients had other diagnoses associated with autism, such as, ADHD (more than 75%), bipolarity (5–8%), depression (4–5%), epilepsy (6–8%). The total of patients that were in use of medication was not different compared baseline (41.8%) and a year later (45.3%). Learning difficulties showed a slight decrease over time. At the beginning, 31.3% of participants reported learning difficulties, with a gradual decrease to 26.4% in the final collection.

Sleep difficulties were present in 44.8% of participants initially, decreasing slightly to 41.5% by the third collection (*p* = 0.021). Functional play, a key area in assessing developmental progress, showed a clear improvement. Initially, 76.1% of participants engaged in functional play, a figure that remained consistent through the second data collection.

Changes in focus, interest, and routine (e.g., repetitive behaviors or resistance to change) showed a decrease over time. Initially, 64.2% of children exhibited these difficulties, but this percentage dropped to 43.4% by the third time point. This suggests that over time, children became less rigid in their routines, possibly reflecting improvement in flexibility and adaptability.

Sensory difficulties were common throughout the study, with 55.2% of participants reporting sensory issues in the first collection, dropping to 47.2% by the third.

There was a negative correlation, for patients between 8–12 years of age, regarding the number of medications used and the number of therapies, that is, the more therapies these patients took, the lower the number of medications in use (*p* = 0.0407, *r* = −0.301).

Patients undergoing psychology intervention, cognitive behavioral therapy, showed improvements in their difficulties with flexibility within 6 months compared to patients who did not receive this intervention. In our study, reductions in repetitive behaviors were considered only when they reflected genuine improvements in flexibility and adaptation, rather than behavioral masking or suppression.

Table 2 shows the CGI and GAF throughout the study. According to the CGI, as answered by caregivers, a reduction in Higher Severity Levels was observed: patients categorized as “Markedly Ill” and “Severely Ill” decreased over time, with no individuals remaining in these categories by the third collection point, suggesting an improvement in the overall clinical status. The percentage of patients in the “Mildly Ill” group increased slightly over time, reaching nearly 40% in the third collection, indicating a shift from more severe to milder symptoms among the group. The proportion of patients rated as “Normal, Not at all ill” and “Borderline Mentally Ill” stayed relatively stable over the three points, with minimal variation, highlighting a consistent population with low symptom severity.

The same questionnaire, the CGI, as answered by the lead therapist, showed that nearly half of the patients are consistently rated as “Borderline Mentally Ill” across the three collection points. This suggests a stable proportion of patients with mild or transient symptoms that may not meet criteria for more severe categories. The “Markedly Ill” and “Severely Ill” categories remain consistent, with 22–26% of patients rated as “Markedly Ill” and around 10% rated as “Severely Ill” across all time points. This stability indicates that a subset of patients is persistently experiencing severe symptoms. Only a very small percentage (1–2%) are rated as “Normal, Not at all Ill,” and there is a slight reduction in the “Mildly Ill” category over time, suggesting limited movement towards complete remission.

The main differences between the two CGI results (one assessed by the responsible and the other by the lead therapist) are evident in the distribution of symptom severity across time points. By a responsible rater, the assessment suggests a trend of improvement over time, with fewer patients in the higher severity categories (“Markedly Ill” and “Severely Ill”) and more patients shifting to the “Mildly Ill” category. This change implies that patients are progressing toward milder symptom stages. However, the lead therapist had no similar improvement trend. The number of patients in the “Markedly Ill” and “Severely Ill” categories remains constant, with no migration to milder levels, suggesting that the lead therapist perceives stability in severe cases, with little overall clinical improvement over time.

For CGI-I, the comparison between responsible raters and lead therapists in assessing patient improvement highlights variations in the perceived rate and extent of clinical gains. Contrary to caregivers, lead therapists reported a sharper increase in “Significant improvement” at 12 months, suggesting that although initially more conservative, therapists ultimately perceived greater clinical progress.

GAF results demonstrated that the proportion of patients in the highest functional categories (GAF 61–100) increases steadily over time, reaching a significant 98.1% by the third collection point. This shift suggests marked improvement in general functioning among the participants, with the majority moving into categories that represent mild or minimal impairment. The percentage of participants in the 51–60 range, which represents moderate impairment, decreases dramatically from 32.8% in the first collection point to just 1.9% in the final point. These GAF results illustrate a clear trend toward improved psychological and social functioning over time, with a majority of participants moving from moderate or serious impairment to mild or near-normal levels of functioning. By the final collection point, nearly all participants are rated in the higher GAF categories (61–100), underscoring the effectiveness of interventions or natural recovery factors. The data suggest both a reduction in the severity of symptoms and an enhancement in daily functioning, reflecting overall positive clinical outcomes across the study period.

Additionally, there was a positive correlation between GAF and intervention time at the clinic (*p* = 0.047, *r* = 0.0478); however, this isolated result does not support causal inferences. There was also a positive correlation between the number of sessions and GAF scores, (*p* = 0.019, *r* = 0.709). Families undergoing family therapy after 6 months and after one year had lower severity levels according to the GAF and CGI.

## 4. Discussion

The study’s findings suggest significant clinical and functional improvements among children with autism across a 12-month period. Most participants showed a shift toward milder symptom categories on the CGI as reported by caregivers, with reductions in severe symptom levels and increases in mild or borderline categories, indicating potential progress in clinical status. General functioning, as measured by the GAF, also improved, with more children reaching higher functional scores at the assessment. Sensory, behavioral, and social characteristics showed reductions over time. Additionally, the data suggested an association between intervention time and functional gains, with lower support needs observed in children engaging in more frequent therapies. These findings should therefore be interpreted as preliminary evidence of potential benefits associated with transdisciplinary interventions.

The predominance of male patients in our study aligns with broader epidemiological trends, where ASD is more frequently diagnosed in males [21]. Research indicates that approximately 41.9% of children with ASD are prescribed psychotropic medications, a rate that reflects the common need to address co-occurring conditions among this population [22]. Additionally, concurrent medication use is prevalent among autistic youth, with over one-third taking two or more medications concurrently for extended periods [23]. Our findings were consistent with these patterns; there was no significant change in medication use across our follow-up period. These trends underscore the importance of integrated public health policies that account for the high prevalence of comorbidities and the ongoing need for both pharmacological and non-pharmacological support. Investments in transdisciplinary services, early access to care, and medication monitoring are critical to ensure safe, equitable, and effective treatment pathways for autistic individuals.

It is essential to emphasize that medication use in ASD primarily targets associated characteristics—such as anxiety, hyperactivity, aggression, and self-injurious behaviors—rather than the core characteristics of autism itself [24]. By managing these experiences, medications can enhance the quality of life for individuals with ASD, often stabilizing behavioral challenges to facilitate engagement in essential non-pharmacological therapies. For instance, antipsychotic medications are sometimes prescribed to help manage severe behavioral challenges in younger children with ASD, thereby supporting their capacity to participate in multidisciplinary interventions tailored to their needs. Beyond pharmacological use, the high prevalence of comorbidities observed in our sample—such as ADHD, mood disorders, and learning difficulties—highlights the complexity of clinical care in ASD. These conditions may significantly influence both treatment response and functional outcomes, underscoring the need for flexible care plans tailored to individual profiles. Importantly, medication and psychosocial interventions should not be viewed as isolated strategies, but rather as potentially complementary approaches. All participants in the study attended at least one individual therapy session, as non-drug therapies play a crucial role in the intervention and support of individuals with ASD. This approach tailors interventions to specific objectives for each patient, considering their age and current needs as well as those of their families. The transdisciplinary setting, characterized by effective team communication, ensures that the goals of each therapy are addressed collaboratively. This structure not only aligns the intervention team but also organizes activities for both the patient and their family, fostering a comprehensive support system.

In this sample, younger patients received a higher number of interventions compared older patients. Typically, younger individuals with ASD require more intensive and varied interventions than older patients. Early intervention is crucial, as it can significantly improve developmental outcomes and reduce the need for additional services later in life [25]. Although intensive interventions have demonstrated positive outcomes in developmental trajectories [26,27], it is important to note that increasing intensity beyond a certain point does not necessarily yield greater benefits, highlighting the importance of balancing therapeutic dose with individual tolerance and engagement [28]. Furthermore, early diagnosis and intervention are linked to better functional outcomes and an improved quality of life [25,29]. It is essential to remember that autism is a lifelong condition, and supports should address each patient’s current needs. While the number of therapies may decrease over time compared to those at the time of diagnosis, this reduction does not imply that the complexity of interventions is any less. Importantly, tailoring intervention intensity to respect each individual’s autonomy, tolerance, and overall well-being is not only clinically sound but also ethically aligned with neurodevelopmental care that values individual differences and self-agency. At the same time, it is important to consider the emotional and logistical burden placed on families, as overly intensive schedules—especially in early years—may contribute to caregiver stress and burnout. Therefore, intervention efforts should be accompanied by ongoing dialogue with families to ensure that therapeutic plans remain sustainable, respectful, and supportive of the family’s well-being.

In our sample, over half of the patients participated in group activities, which are well-documented in the literature as beneficial for individuals with autism, particularly for enhancing social skills [30]. For instance, a review of 15 studies on shared social activity-based interventions—such as interest-based games, music, and theater—found positive social outcomes for children on the autism spectrum interacting with typically developing peers [31]. Physical activities that can be conducted in groups, such as yoga, also show promise as support for autistic patients [32]. However, implementing group activities for individuals with ASD poses challenges, particularly in areas such as social communication [33], sensory sensitivities [34], lack of awareness, and accessibility barriers. Addressing these challenges requires a translational approach, such as the one employed in our study, which includes educating peers and staff, modifying physical environments, and creating inclusive and supportive group activities.

Group activities and social skills training provide structured environments where individuals with ASD can practice social interactions and develop relationships. These group settings offer essential opportunities for peer modeling and feedback, both crucial for fostering social competence. Moreover, participating in group activities can alleviate feelings of isolation and foster a sense of community and belonging [35]. Access to group activities may be limited due to service availability and resource constraints such as specialized staff and caregiver time.

The relationship between socioeconomic status and ASD is complex and multifaceted. Various studies have highlighted how SES influences the diagnosis, access to services, and overall quality of life for individuals with autism. Research indicates that children from higher socioeconomic backgrounds are more likely to receive an early identification of autism. This is often attributed to better access to health care services, greater awareness, and the ability to afford private evaluations [36]. Access to therapeutic and educational services is crucial for the development of children with autism. However, socioeconomic disparities significantly affect this access. Families with higher incomes can afford specialized therapies, private schooling, and additional support services that may not be available through public systems [37]. These disparities are not merely individual or circumstantial, but reflect broader structural inequalities embedded in health, education, and social policy systems. A lack of trained professionals, early screening programs, and consistent intervention pathways in resource-limited regions highlights the need to address systemic gaps in order to promote equity in ASD diagnosis and care.

The results from the CGI and GAF assessments reveal differing perspectives on patient progress between responsible raters (often family members or caregivers) and lead therapists for patients with autism. This divergence aligns with findings in autism research, where variations in the perceived progress and daily functioning of individuals with autism are common between family members and clinical professionals. Caregivers may observe and value broader improvements in behavior and engagement across varied settings, while therapists may focus on more specific, therapy-centered skills and the stability of specific characteristics. These differences may also be linked to the concept of experiential validation, whereby caregivers’ lived experiences influence how they interpret and assign meaning to change.

In the CGI data, caregiver raters showed a greater tendency to rate patients as “Much Improved” or “Improved,” indicating their perception of significant overall improvement. This may reflect the family members’ daily interactions with the individual, which allow them to observe incremental progress in social engagement, independence, and adaptability. Literature suggests that caregivers often report positive changes in adaptive functioning and social interaction in familiar environments, possibly influenced by their proximity to the individual’s day-to-day challenges and milestones [38,39]. Family members might thus interpret gains in communication, emotional regulation, and behavior management as substantial improvements, particularly if these changes facilitate more harmonious family interactions.

In contrast, lead therapists were more conservative in their improvement ratings, particularly in assigning high GAF scores. This cautious outlook is common among clinicians, who may set higher thresholds for meaningful change, often looking for sustained progress in therapy-specific goals such as structured communication, social skills, and behavioral interventions [40,41]. Therapists, working with patients in structured settings, may observe and assess improvements within narrower contexts, focusing on progress directly related to intervention goals rather than broader functioning. For example, while a caregiver might view a reduction in disruptive behaviors as a major improvement, a therapist may only rate this change as “Slightly Improved” if the improvement is inconsistent or limited to specific settings [42].

The GAF results further illustrate these differences in perspective, showing that caregiver raters consistently view more individuals as functioning at higher levels over time. The majority of patients, by the final assessment, scored in the GAF range of mild impairment or better according to caregivers, compared to the more conservative therapist ratings. This aligns with findings in autism research suggesting that clinicians, who emphasize clinical stability, may view functional improvement more cautiously, potentially underestimating changes that are context-dependent [25]. Additionally, caregivers’ optimism might be influenced by observing improvements in unstructured settings, which could enhance their perception of a child’s functional abilities, even if such changes are not as evident in therapeutic sessions [43,44]. These contrasting perspectives underscore the importance of incorporating multiple viewpoints when interpreting outcomes, as each captures different aspects of the individual’s functioning and adaptation. These findings reflect the need for a multi-perspective approach in evaluating functional improvements in autism, as both caregiver and therapist insights contribute unique and valuable views. Understanding these perspectives is critical, as it can help bridge gaps between therapy goals and real-world functionality, ensuring that therapeutic interventions are more tailored to both clinical and everyday needs.

This study presents limitations that should be acknowledged. First, the absence of a control group—due to the real-world clinical setting—limits the ability to establish causal inferences, while the wide and heterogeneous age range of participants (2 to 16 years) may have introduced variability in responses and reduced the generalizability of the findings. The fact that socioeconomic factors were analyzed only descriptively may have limited a deeper understanding of their influence on outcomes. Regarding the questionnaires used, these instruments were selected due to their wide clinical use and feasibility in routine care. Although studies such as Livingston et al. (2017) [45] support their relevance and clinical utility in assessing outcomes in autistic individuals, particularly in naturalistic settings, their ecological validity and sensitivity to the lived experiences of neurodivergent populations remain subject to debate. The study did not apply multivariate adjustments to control for potential confounding variables, which limits causal inference. Nonetheless, the longitudinal design with repeated standardized assessments contributes to the internal consistency of the observed outcomes. Although comorbidities such as ADHD and mood disorders were prevalent in the sample, no stratified analysis was conducted to explore their specific interaction with intervention outcomes. Additionally, the study did not include direct input from the autistic community, incorporating participatory models involving autistic individuals may enhance inclusivity. Future research may benefit from incorporating autistic perspectives to help guide intervention development and evaluation, aligning with calls for more inclusive and mutual understanding in support approaches [46].

Despite these limitations, the study offers significant translational value by bridging clinical practice and research in a real-world setting. A key innovation is the implementation of a flexible, transdisciplinary, and family-centered model, where intervention phases are dynamically tailored to each child’s evolving needs. The integration of culturally relevant group activities adds ecological validity and supports generalization across contexts. Moreover, the inclusion of both caregiver and therapist assessments provides a more nuanced understanding of perceived outcomes, enhancing the study’s originality and practical relevance.

## 5. Conclusions

In conclusion, this study suggests potential improvements in both the clinical status and overall functionality of children with ASD following a 12-month period of transdisciplinary interventions. There was a reduction in severe symptoms and a shift toward milder symptom categories as reported by caregivers, along with observed gains in daily functionality according to the GAF scale. Differences between caregiver and therapist assessments highlight the importance of considering multiple perspectives when evaluating progress, as each group captures distinct aspects of the child’s functionality and adaptation in daily and therapeutic contexts. The observed association between therapy intensity and developmental progress, particularly in younger patients, points to the potential value of early and integrated interventions. This study reinforces the effectiveness of transdisciplinary interventions in supporting the development and quality of life of individuals with ASD and suggests that including collaborative evaluations between caregivers and professionals can offer a more comprehensive view of the needs and progress of these patients. The observed improvements should be interpreted in the context of a structured, clinic-based intervention model grounded in transdisciplinary collaboration and tailored to each individual’s evolving profile.

This article offers new contributions to the literature in several key areas. Firstly, the use of a one-year longitudinal methodology focused on transdisciplinary and transdisciplinary interventions provides a unique perspective on how integrated approaches can impact the development of children and adolescents with ASD. The dynamic intervention model, which allows progression through intervention phases based on individual responses, is relatively underexplored in longitudinal ASD intervention studies. Additionally, the application of a structured methodology, which fosters continuous communication among professionals from different fields, presents an innovative form of transdisciplinarity. This could improve practices in ASD support centers and influence public health policies by promoting evidence-based, patient-centered care models. Lastly, the study highlights the emotional and social impacts of integrated therapies, emphasizing the importance of emotional bonds and family involvement in the therapeutic process. This aspect, which focuses on the relationship between therapy and emotional connections, is relatively underexplored in the existing ASD literature, which often prioritizes more rigid behavioral approaches. The study’s emphasis on relational, humanized interventions could encourage a shift in the support paradigm for ASD.

Future research should explore the long-term outcomes of transdisciplinary models in more representative populations. Studies should also incorporate formal controls for potential confounding variables to strengthen the validity of findings. Additionally, policy efforts should focus on expanding access to culturally contextualized, team-based care structures that support family engagement, professional collaboration, and individualized therapeutic planning.

## Figures and Tables

**Table 1:** Descriptive characteristics of the study population.

Variable	Baseline *n* = 67	6 Months *n* = 58	12 Months *n* = 53	*p*
ABEP				0.81
A	43 (64.2)	35 (60.3)	32 (60.4)	
B	23 (34.3)	22 (37.9)	21 (39.6)	
C	1 (1.5)	1 (1.7)	-	
D	-	-	-	
Age	9.00 ± 3.92	8.00 ± 3.66	8.32 ± 3.91	0.28
Ethnicity				0.98
White	57 (85.1)	48 (82.8)	43 (81.1)	
Grey-brown	4 (6.0)	4 (6.9)	4 (7.5)	
Black	6 (9.0)	6 (10.3)	6 (11.3)	
Gender				0.78
Female	13 (19.4)	13 (22.4)	9 (17.0)	
Male	54 (80.6)	45 (77.6)	44 (83.0)	
Pregnancy complications				0.96
Yes	5 (7.5)	4 (6.9)	4 (7.5)	
No	62 (92.5)	54 (93.1)	49 (92.5)	
Use of medication during pregnancy				0.87
Yes	12 (17.9)	10 (17.2)	9 (17.0)	
No	55 (82.1)	48 (82.8)	44 (83.0)	
Baby delivery				0.89
Normal delivery	15 (22.4)	11 (19.0)	10 (18.9)	
C-section	52 (77.6)	47 (81.0)	43 (81.1)	
Delivery complications				
Yes	7 (10.4)	6 (10.3)	6 (11.3)	0.98
No	60 (89.6)	52 (89.7)	47 (88.7)	

Data are presented as absolute frequencies (*n*) and percentage (%). Percentages were calculated based on the total number of respondents for each time point, as indicated in the column headings. A dash (-) indicates that no responses were recorded for that category. Categorical variables were compared across time points using Pearson’s chi-square test, while the continuous variable (age) was analyzed using repeated-measures ANOVA. Analyses were conducted using SPSS software, version 25.0 (SPSS Inc., Chicago, IL, USA).

**Table 2:** Clinical Global Impression (CGI) and Global Assessment Functioning (GAF) descriptive results.

Variable	Baseline *n* = 67	6 Months *n* = 58	12 Months *n* = 53	*p*
CGI-S (Caregivers)				0.067
Normal, not at all Ill	4 (6.0)	3 (5.2)	3 (5.7)	
Borderline Mentally Ill	13 (19.4)	14 (24.1)	12 (22.6)	
Mildly Ill	20 (29.9)	18 (31.0)	21 (39.6)	
Moderately Ill	18 (26.9)	17 (29.3)	17 (32.1)	
Markedly Ill	11 (16.4)	5 (8.6)	-	
Severely Ill	1 (1.5)	1 (1.7)	-	
Extremely severe Ill	-	-	-	
CGI-S (Lead Therapist)				0.413
Normal, not at all Ill	1 (1.5)	1 (1.7)	1 (1.9)	
Borderline Mentally Ill	33 (49.3)	27 (46.6)	24 (45.3)	
Mildly Ill	9 (13.4)	7 (12.1)	5 (9.4)	
Moderately Ill	3 (4.5)	3 (5.2)	3 (5.7)	
Markedly Ill	15 (22.4)	14 (24.1)	14 (26.4)	
Severely Ill	6 (9.0)	6 (10.3)	6 (11.3)	
Extremely severe Ill	-	-	-	
CGI-I (Caregivers)				0.027
Significant improvement	-	6 (10.3)	21 (39.6)	
Improvement	-	17 (29.3)	-	
Slight improvement	-	25 (43.1)	22 (41.5)	
No change	-	10 (17.2)	10 (18.9)	
CGI-I (Lead Therapist)				0.004
Significant improvement	-	18 (31.0)	42 (79.2)	
Improvement	-	26 (44.8)	-	
Slight improvement	-	10 (17.2)	9 (17.0)	
No change	-	2 (3.4)	2 (3.8)	
GAF				0.512
91–100	1 (1.5)	2 (3.4)	2 (3.8)	
81–90	5 (7.5)	4 (6.9)	3 (5.7)	
71–80	18 (26.9)	18 (31.0)	17 (32.1)	
61–70	16 (23.9)	15 (25.9)	30 (56.6)	
51–60	22 (32.8)	16 (27.6)	1 (1.9)	
41–50	4 (6.0)	2 (3.4)	-	
21–30	-	-	-	
11–20	-	-	-	
1–10	-	-	-	

CGI-S: Clinical Global Impression—Severity, CGI-I: Clinical Global Impression—Improvement, GAF: Global Assessment Functioning. Data are presented as absolute frequencies (*n*) and percentage (%). Percentages were calculated based on the total number of respondents for each time point, as indicated in the column headings. A dash (-) indicates that no responses were recorded for that category. Categorical variables were compared across time points using the Pearson’s chi-square test. Analyses were conducted using SPSS software, version 25.0 (SPSS Inc., Chicago, IL, USA).

## Data Availability

The data presented in this study are not publicly available due to privacy and ethical restrictions.

## Abbreviations

The following abbreviations are used in this manuscript:

ASD	Autism Spectrum Disorder
CGI	Clinical Global Impression
GAF	Global Assessment of Functioning
ABC	Aberrant Behavior Checklist
DSM	Diagnostic and statistical Manual of Mental Disorders
DSM-5-TR	Diagnostic and Statistical Manual of Mental Disorders, Fifth Edition, Text Revision
ADHD	Attention Deficit Hyperactivity Disorder
OCD	Obsessive–Compulsive Disorder
ODD	Oppositional Defiant Disorder
CCEB	Brazilian Economic Classification Criterion
ABEP	Brazilian Association of Research Companies

## Appendix A

**Table A1:** Prevalence and frequency per week of patients by individual and group therapy by modality.

Variable	Baseline *n* = 67	6 Months *n* = 58	12 Months *n* = 53
Individual therapy	67 (100)	58 (100)	53 (100)
Group therapy	40 (59.7)	36 (62.1)	29 (54.7)
Individual therapy by modality			
*Speech Therapy*	56 (83.6)	48 (82.8)	43 (81.1)
Once a week	32 (47.8)	27 (46.6)	25 (47.2)
Twice a week	23 (34.3)	21 (36.2)	18 (34.0)
Three times a week	1 (1.5)	-	-
*Psychology*	52 (77.6)	45 (77.6)	41 (77.4)
Once a week	46 (68.7)	40 (69.0)	37 (69.8)
Twice a week	6 (9.0)	5 (8.6)	4 (7.5)
*Occupational Therapy*	34 (50.7)	29 (50.0)	26 (49.1)
Once a week	29 (49.3)	24 (41.4)	21 (39.6)
Twice a week	5 (7.5)	5 (8.6)	5 (9.4)
*Psychopedagogy*	15 (22.4)	13 (22.4)	10 (18.9)
*Family Therapy*	26 (38.8)	23 (39.7)	20 (37.7)
Group therapy by modality			
Capoeira	3 (4.5)	2 (3.4)	2 (3.8)
Judo	7 (10.4)	9 (15.5)	6 (11.3)
Theater	10 (14.9)	9 (15.5)	10 (18.86)
Psychomotricity	5 (7.5)	3 (5.2)	3 (5.7)
Music	10 (14.9)	6 (10.3)	5 (9.4)
Dance	3 (4.5)	3 (5.2)	2 (3.8)
Social Skills Group	12 (17.9)	7 (12.0)	8 (15.0)
Yoga	7 (10.4)	8 (13.8)	6 (11.3)

Data are presented as absolute frequencies (*n*) and percentage (%). Percentages were calculated based on the total number of respondents for each month, as indicated in the column headings. A dash (-) indicates that no responses were recorded for that category.
